# Assessment of appetite using health-care-professional-reported SNAQ

**DOI:** 10.1016/j.jnha.2025.100693

**Published:** 2025-10-06

**Authors:** Jérémy Raffin, Stéphane Gerard, Morgane Naudy, Marion Meras, Yves Rolland

**Affiliations:** a IHU HealthAge, Institut du Vieillissement, Gérontopôle de Toulouse, Centre Hospitalo-Universitaire de Toulouse, 37 Allées Jules Guesde, 31000 Toulouse, France; b Hopital Purpan, Centre Hospitalo-Universitaire de Toulouse, France; c SMR Onco-gériatrique et Neurolocomoteur, Hôpital Garonne, Centre Hospitalo-Universitaire de Toulouse, France; d CERPOP UMR 1295, University of Toulouse III, Inserm, UPS, Toulouse, France

**Keywords:** Appetite, Nutrition, Questionnaire, Anorexia

## Abstract

**Background:**

Appetite loss is a major concern in various clinical contexts but the self-assessment of appetite might be compromised in some populations such as in individuals with dementia. The use of surrogate responders might be useful but no study so far has investigated whether hetero-administrated questionnaires might be valid tools to assess appetite.

**Methods:**

Forty men and women aged 66–97 years, without dementia, were recruited from the Medical care and Rehabilitation Unit of the Toulouse University Hospital, France. Within the same day, patients’ appetite was assessed using the SNAQ, a 4-answers questionnaire that was administrated separately to the patients as well as to two certified nursing assistant (CNA) surrogate responders answering on behalf of the patients. Intraclass correlation coefficients (ICC) as well as Kappa’s and Gwet’s AC1 coefficients were computed to test the agreement between the patients and each surrogate assessor and between the two surrogate assessors.

**Results:**

When comparing patients versus surrogate responders’ SNAQ scores, ICC (95% Confidence Interval (CI)) showed weak to moderate correlations, ranging from 0.087 (−0.224, 0.384) to 0.519 (0.254, 0.713). The rate of correct categorization was 65.0–67.5 % and Kappa’s and Gwet’s coefficients ranged from 0.300 (0.006, 0.594) to 0.360 (0.082, 0.638), indicating poor agreements between patient and surrogates. When comparing the surrogate responders with each other, the percentage of similar diagnosis was 82.5% with corresponding Kappa’s and Gwet’s coefficients of 0.650 (0.417, 0.883) and 0.652 (0.406, 0.898), indicating good agreements among surrogate raters.

**Conclusion:**

We found weak-to-moderate coefficients for the SNAQ used as a hetero-administrated questionnaire. Further investigation across diverse clinical settings and using larger samples are needed to provide further insights to our preliminary observations.

## 1 Introduction

Appetite loss represents a major concern in clinical populations such as individuals with cancer [1] or older adults with multiple comorbidities including those who live in nursing homes [2]. Appetite loss can reach a prevalence of more than 30% in institutionalized people and has been associated with an increased risk of mortality and various adverse outcomes such as sarcopenia and frailty [3,4]. Hence, screening appetite loss represents an important part of the preventive medical care with self-reported questionnaires and food intake recording being the most commonly used methods [5]. Yet, in some contexts, the use of self-administrated questionnaires may be compromised by the presence of conditions such as cognitive impairment. In these cases, the help of a surrogate responder (i.e., hetero-administrated questionnaire) such as a family proxy or a medical staff member would represent a quicker and simpler alternative to food intake recording. The simplified nutritional appetite questionnaire (SNAQ), the short version of the council on nutrition appetite questionnaire (CNAQ) [6], has been shown to be a valid and reliable self-reported questionnaire in community-dwelling [7] and institutionalized older adults [8], and significantly predicts poor health outcomes in hospitalized older adults [9]. However, previous works have highlighted its limitation in using this tool in people with dementia [10]. Some authors have suggested that this questionnaire may be used as a hetero-administrated questionnaire [11], although no study so far has explicitly tested this hypothesis. In the present study, we examined the validity of the SNAQ when used as a hetero-administrated questionnaire in a sample of hospitalized adults without dementia.

## 2 Methods

The present study was registered and conducted at the Toulouse University Hospital, France (reference: RnIPH 2025-119, number: HDH 26461499). It was covered by the reference methodology MR-004 of the National Commission for Informatics and Liberties ((CNIL) number: 2206723 v 0), validated by the data protection officer, and complied with the General Data Protection Regulation. According to French Ethics and Regulatory law, prospective studies based on the exploitation of usual care data are not required to be submitted to an ethics committee. Patients were informed that their codified data were used for the study; written consent was not required.

### 2.1 Subjects

During August and September 2024, 40 subjects were recruited from the Medical care and Rehabilitation Unit of the Toulouse University Hospital.Reasons of hospitalization were rehabilitation after acute care such as hip fracture surgery, pneumonia, heart failure, chemotherapy for cancer, or falls. They were included in this study if they had a Mini Mental State Examination score of 18 or above and if they had been admitted to the department for at least 7 days. Basic demographic data, including age, sex, bodyweight, height, activity of daily living (ADL) score [12], reason for admission and total daily calorie intake were collected for each participant.

### 2.2 Appetite assessment

The SNAQ is a short version of the CNAQ. It includes 4 questions about appetite, satiety, food tastes, and meal frequency. For each question, the patient responds on a Likert-type scale with 5 possible answers. The SNAQ score can range from 4 to 20 and a score ≤14 indicates appetite loss with a clinically meaningful risk of 5% weight loss within six months [6].

The appetite of each patient was evaluated three times during the same day: first by the patient him/herself (auto-administrated questionnaire) and then by two different certified nursing assistants (CNA) (who could be different from one patient to another) who to took care of the patients for at least one week (hetero-administrated questionnaire). These professionals were deemed to be the best to take on this role as they were those who knew the patients’ eating habits the most since they accompanied them at mealtimes. Staff members where blinded to the patients answers when completing the SNAQ. Each SNAQ administration was performed under the supervision of a dietician. In addition to the SNAQ administration, appetite was also assessed on a visual analogue scale (VAS) ranging from 0 to 100. Both the patients and CNA were asked to answer the following question “How would you rate your appetite / the appetite of the patient? Please indicate on the scale the value that fit the best. 0 corresponds to no appetite at all, 100 indicates a very good appetite”. Data on the daily calorie and protein intake of each patient were also collected.

### 2.3 Statistical analyses

Participants’ characteristics were provided as median and interquartile ranges (IQR) for continuous variables and numbers with percentages for categorical variables. The strength of the agreements for the SNAQ total score and the score for each question, as well as for the VAS ratings were assessed using intraclass correlation coefficients (ICC). Since the surrogate responders were randomly assigned to each patient and could be different from one patient to another, a single-measure one-way random-effect model was used for comparing the scores of the patients versus those of the surrogate responders, while a two-way random effect model was used for comparing the scores of the surrogate responders with each other [13]. Cohen’s Kappa and Gwet’s AC1 coefficients [14] were also computed using the SNAQ score as dichotomous variable defined as having or not a score ≤14. Each coefficient was computed while comparing the patients’ scores with each of the two medical staff members, and when comparing the two medical assistant scores with each other (without considering the patient’s scores). ICC, Kappa’s and Gwet’s coefficient values of 0.6 and above were considered as good agreement [15]. In addition, Spearman correlations, conducted separately for the patients, the first, and the second surrogates, were performed in order to test whether the SNAQ total scores correlated with the patients’ total daily calorie intake as well as with the VAS ratings. Moreover, estimated energy requirements (EER) were computed for each patient based on validated equations [16] and were compared with their actual daily calorie intake. Patients were classified as meeting or not meeting their EER (daily calorie intake ≥ EER vs < EER). Chi-squared tests were then performed to assess whether the proportion of patients diagnosed with appetite loss (SNAQ score ≤14) differed in patients who met their EER compared to those who did not met their EER. All analyses were performed using the software SPSS statistics and R with a significance threshold set at p<0.05.

## 3 Results

The sample included in this study was composed of 62.5% (25/40) females and the median (interquartile range-IQR) age was 81.5 (77–86) years, ranging from 66–97. The median (IQR) Katz’s Activity Daily Living (ADL) score was 4.1 (3.3–5.5), and 55% (22/40) of the participants had a SNAQ score ≤14, indicating a loss of appetite. Further details on the patients’ characteristics are provided in Table 1.

**Table 1:** Characteristics of the participants.

	Total		SNAQ score > 14		SNAQ score ≤14	
	Sample size	Statistic	Sample size	Statistic	Sample size	Statistic
Age (years)	40	81.5 (77–86)	18	81.5 (77–85)	22	82 (77–87)
Females	40	25 (62.5%)	18	13 (72.2%)	22	12 (54.5%)
BMI (kg/m²)	40	40 (100%)	18	18 (100%)	22	22 (100%)
Underweight (≤18.5 kg/m²)	40	2 (5%)	18	1 (5.6%)	22	1 (4.5%)
Normal weight (18.5–24.9 kg/m²)	40	15 (37.5%)	18	5 (27.8%)	22	10 (45.5%)
Overweight (25−29.9 kg/m²)	40	13 (32.5%)	18	8 (44.4%)	22	5 (22.7%)
Obesity (≥30 kg/m²)	40	10 (25%)	18	4 (22.2%)	22	6 (27.3%)
ADL score (0−6)	40	4.1 (3.3–5.5)	18	5 (3.5–5.8)	22	3.6 (2.8–5)
Daily calorie intake (kcal/d)	40	1875 (1725–2177.5)	18	1950 (1730–2240)	22	1797.5 (1720–2145)
Females	25	1766 (1720–1980)	13	1880 (1730–2000)	12	1763 (1614–1837.5)
Males	15	2180 (1790–2300)	5	2312 (2175–2320)	10	2162.5 (1790–2250)
Daily protein intake (g/kg/d)	40	1.1 (0.9–1.4)	18	1.2 (1.1–1.4)	22	1 (0.9–1.4)
Females	25	1.1 (0.9–1.3)	13	1.2 (1.1–1.4)	12	1 (0.9–1.2)
Males	15	1.1 (0.9–1.5)	5	1.2 (1.1–1.2)	10	1 (0.9–1.5)
SNAQ score (4−20)	40	14 (12.5–17)	18	17 (16–18)	22	13 (11–14)
VAS	40	50 (50–75)	18	80 (60–90)	22	50 (40–50)

Statistics are provided as median (IQR) for continuous variables and numbers (%) for categorical variables. ADL: activities of daily living; BMI: body mass index; SNAQ: simplified nutritional appetite questionnaire; VAS: visual analog scale.

ICC (95% Confidence Interval (CI)) for the SNAQ total score and for each question score as well as for the VAS ratings are provided in Table 2 and ranged from 0.067 (−0.254, 0.371) to 0.545 (0.290, 0.729), indicating weak-to-moderate agreements. When considering the SNAQ as dichotomous variable, the percentage of patients correctly categorized by the first and second raters was 67.5% and 65%, with corresponding Kappa’s coefficients (95% CI) of 0.360 (0.082, 0.638) and 0.300 (0.006, 0.594), and corresponding Gwet’s coefficients (95% CI) of 0.350 (0.047, 0.654) and 0.302 (−0.008, 0.611), respectively, as shown in Table 3. The percentage of similar diagnosis between the two surrogate raters was 82.5% with corresponding Kappa’s and Gwet’s coefficients of 0.650 (0.417, 0.883) and 0.652 (0.406, 0.898), respectively. Spearman’s correlation analyses demonstrated that the patients’ total daily calorie intake was correlated with the surrogates’ SNAQ total scores (rho = 0.43, p = 0.006 and rho = 0.45, p = 0.004) but not with the patients’ scores (rho = 0.26, p = 0.108). In addition, strong correlations were observed between the SNAQ and the VAS scores with rho coefficients of 0.868, 0.850 and 0.841 for the patients, the first surrogate responder, and the second one, respectively (all p < 0.001). Chi-square tests indicated that the proportion of patients categorized with appetite loss based on the self-administrated questionnaire was significantly higher in patients who met their EER compared to those who did not meet their EER (84.6% vs 40.7%, p = 0.009). Such associations were not found significant when using the hetero-administrated questionnaires (61.5% vs 33.3%, p = 0.91 for surrogate responder 1 and 61.5% vs 44.4%, p = 0.311 for surrogate responder 2).

**Table 2:** Intraclass correlation coefficients for the SNAQ and the VAS scores.

Variable	Comparison	ICC (95% CI)	p
SNAQ total score	Patient vs Surrogate 1	0.435 (0.149, 0.654)	0.002
SNAQ total score	Patient vs Surrogate 2	0.519 (0.254, 0.713)	<0.001
SNAQ total score	Surrogate 1 vs Surrogate 2	0.537 (0.278, 0.725)	<0.001

SNAQ-Q1 score	Patient vs Surrogate 1	0.290 (-0.017, 0.548)	0.032
SNAQ-Q1 score	Patient vs Surrogate 2	0.420 (0.131, 0.644)	0.003
SNAQ-Q1 score	Surrogate 1 vs Surrogate 2	0.501 (0.229, 0.701)	<0.001

SNAQ-Q2 score	Patient vs Surrogate 1	0.459 (0.178, 0.671)	0.001
SNAQ-Q2 score	Patient vs Surrogate 2	0.394 (0.101, 0.625)	0.005
SNAQ-Q2 score	Surrogate 1 vs Surrogate 2	0.545 (0.290, 0.729)	<0.001

SNAQ-Q3 score	Patient vs Surrogate 1	0.376 (0.079, 0.612)	0.007
SNAQ-Q3 score	Patient vs Surrogate 2	0.208 (-0.104, 0.484)	0.094
SNAQ-Q3 score	Surrogate 1 vs Surrogate 2	0.362 (0.057, 0.605)	0.011

SNAQ-Q4 score	Patient vs Surrogate 1	0.087 (-0.224, 0.384)	0.292
SNAQ-Q4 score	Patient vs Surrogate 2	0.429 (0.142, 0.650)	0.002
SNAQ-Q4 score	Surrogate 1 vs Surrogate 2	0.067 (-0.254, 0.371)	0.342

VAS score	Patient vs Surrogate 1	0.317 (0.012, 0.568)	0.021
VAS score	Patient vs Surrogate 2	0.369 (0.072, 0.607)	0.008
VAS score	Surrogate 1 vs Surrogate 2	0.503 (0.229, 0.703)	<0.001

CI: confidence interval; ICC: intraclass correlation coefficient; Q: question; SNAQ: simplified nutritional appetite questionnaire; VAS: visual analog scale.

**Table 3:** Gwet’s and Kappa’s coefficients for dichotomous SNAQ score.

Variable	Comparison	% similar diagnosis	Gwet AC1 (95% CI)	p	Kappa (95% CI)	p
SNAQ score ≤14	Patient vs Surrogate 1	67.5	0.350 (0.047, 0.654)	0.025	0.360 (0.082, 0.638)	0.019
SNAQ score ≤14	Patient vs Surrogate 2	65.0	0.302 (-0.008, 0.611)	0.056	0.300 (0.006, 0.594)	0.057
SNAQ score ≤14	Surrogate 1 vs Surrogate 2	82.5	0.652 (0.406, 0.898)	<0.001	0.650 (0.417, 0.883)	<0.001

CI: confidence interval; SNAQ: simplified nutritional appetite questionnaire.

## 4 Discussion

In the present study, we investigated whether the SNAQ can be used by health care professionals as a hetero-administrated questionnaire, that is, by asking a surrogate responder to answer on behalf of the patient, in a sample of 40 patients without dementia admitted in Medical care and Rehabilitation Unit. Our results from this preliminary study revealed weak-to-moderate agreement coefficients, indicating a need for more in-depth investigations.

The SNAQ is a self-administrated questionnaire that was developed to quickly assess appetite in community-dwelling and nursing home older adults. It includes 4 questions that ask about appetite, satiety, food tastes, and meal frequency on a five-level Likert scale [8]. The lack of agreement when comparing the answers obtained from the patients with those of the CNA may be due to the subjective nature of appetite that renders it difficult to assess on behalf of someone else. Consistently, previous work on other subjective outcomes such as pain, also reported poor agreements between patients and surrogate responders [17]. Interestingly, our results revealed that even the question about meal frequency (Question #4) [6], which is rather an objective outcome, did not provide better ICC scores compared to the other questions. Yet, previous studies conducted in patients with dementia have indicated that information about objective outcomes such as physical performance, medical or lifestyle history, were valid when obtained from surrogate responders [[18], [19], [20]]. Further work is required to better understand the reasons for the lack of consistency observed in our study. Possibly, stronger agreements would be obtained in long term clinical settings where healthcare professionals have longer time to know the patients. In this regard, family proxies in the community might also provide more accurate assessments than health care professionals in assessing patients’ appetite. Caregivers are used to assess food intake, but training on appetite and its assessment is probably insufficient. Of note, the patients’ daily total calorie intake was correlated with the SNAQ scores obtained from the CNA but not with those reported by the patients. Conversely, EER status was associated with appetite loss diagnosed from the patients’ questionnaires but not from the surrogates’ questionnaires. It is nevertheless encouraging to note that surrogates shared 82.5% of common diagnosis and displayed good Kappa’s and Gwet’s coefficients (0.65 for both) when comparing their responses with each other. This indicates that healthcare professionals share a similar perception of patients’ appetite, although it seems to differ from that of the patients themselves. Also the responses between the SNAQ and the VAS were also consistent within patients and surrogate raters with correlations coefficients above 0.8.

The main strength of this study is its novelty since it is the first investigation, to our knowledge, that statistically evaluated the use of the SNAQ as a hetero-administrated questionnaire. This work, however, remains limited by its small sample size and the fact patients from medium-stay units were included which could have impeded the surrogate responders to get sufficient knowledge on patients’ appetite before answering the questionnaires. Another limitation might be the reduced number of questions included in the SNAQ; using the CNAQ, the full version of the questionnaire that contains 8 questions [6], may have provided more accurate assessment of appetite and perhaps stronger agreements between patients and surrogate responders. However, longer questionnaires may be less feasible to use in clinical settings.

In conclusion, our study results indicate weak-to-moderate agreements when using the SNAQ as a hetero-administrated questionnaire. However, good agreements were obtained when comparing the surrogate responders with each other. Further work using large samples across populations from various clinical and non-clinical settings, and using different tools to assess appetite such as questionnaires, visual analogue scales, food intake recording or biomarker measurement, are needed to support our preliminary findings and clarify whether or not appetite can effectively be assessed from surrogate proxies.

## CRediT authorship contribution statement

JR and YR conceptualized and designed the present study. SG, MN, and MM, collected the data. JR analysed the data, performed the statistical analyses, produced the tables and wrote the manuscript. All authors interpreted the data, critically revised the manuscript for intellectual content, read and approved the final manuscript before publication.

## Declaration of Generative AI and AI-assisted technologies in the writing process

No AI was used for this work.

## Declaration of competing interest

The authors declare that they have no known competing financial interests or personal relationships that could have appeared to influence the work reported in this paper.
